# Antioxidant properties of fermented soymilk and its anti-inflammatory effect on DSS-induced colitis in mice

**DOI:** 10.3389/fnut.2022.1088949

**Published:** 2023-01-06

**Authors:** Yijiao Sun, Jingting Xu, Huiyan Zhao, Yue Li, Hui Zhang, Baichong Yang, Shuntang Guo

**Affiliations:** ^1^Beijing Key Laboratory of Plant Protein and Cereal Processing, College of Food Science and Nutritional Engineering, China Agricultural University, Beijing, China; ^2^Pony Testing International Group Co., Ltd., Beijing, China

**Keywords:** lactic acid bacteria, fermented soymilk, anti-inflammatory effect, antioxidant effect, physiologically active ingredients

## Abstract

Lactic acid-fermented soymilk as a new plant-based food has aroused extensive attention because of its effects on nutrition and health. This study was conducted to delve into the antioxidative and anti-inflammatory activities of lactic acid-fermented soymilk. To elucidate the key factors that affect the antioxidant properties of fermented soymilk, the strains and preparation process were investigated. Findings show that the fermented soymilk prepared using hot-water blanching method (BT-80) demonstrated a better antioxidant activity than that using conventional method (CN-20). Besides, a huge difference was observed among the soymilks fermented with different strains. Among them, the YF-L903 fermented soymilk demonstrated the highest ABTS radical scavenging ability, which is about twofold of that of unfermented soymilk and 1.8-fold of that of L571 fermented soy milk. *In vitro* antioxidant experiments and the analysis of H_2_O_2_-induced oxidative damage model in Caco-2 cells showed that lactic acid-fermentation could improve the DPPH radical scavenging ability, ABTS radical scavenging ability, while reducing the content of reactive oxygen species (ROS) and malondialdehyde (MDA) in Caco-2 cells induced by H_2_O_2_, and increasing the content of superoxide dismutase (SOD). Consequently, cells are protected from the damage caused by active oxidation, and the repair ability of cells is enhanced. To identify the role of fermented soymilk in intestinal health, we investigate its preventive effect on dextran sodium sulfate-induced colitis mouse models. Results revealed that the fermented soymilk can significantly improve the health conditions of the mice, including alleviated of weight loss, relieved colonic injury, balanced the spleen-to-body weight ratio, reduced the disease index, and suppressed the inflammatory cytokines and oxidant indexes release. These results suggest that YF-L903 fermented soymilk is a promising natural antioxidant sources and anti-inflammatory agents for the food industry. We believe this work paves the way for elucidating the effect of lactic acid-fermented soymilk on intestinal health, and provides a reference for the preparation of fermented soymilk with higher nutritional and health value.

## 1. Introduction

Soymilk, as a traditional plant protein beverage in China, is rich in soybean nutrients (e.g., protein and fat) and a variety of physiologically active substances (e.g., polyphenols, isoflavones, saponins, and phytosterols) (1, 2). Previous studies have found that these physiologically active ingredients play a significant role in the healthy functions of soymilk and other soy foods, and can prevent a variety of chronic diseases, including cardiovascular diseases, insulin resistance, breast cancer, and immune dysregulation (3). In recent years, with the rise of plant-based foods (4–6), fermented soymilk has attracted extensive attention because of its nutritionally and physiologically active components changes and its consequent enhanced health effects during fermentation (7–10). Fermented soymilk prepared using special *Lactobacillus fermentum* obtained from Xinjiang yak yogurt, Chongqing kimchi and Taiwan fermented cabbage, such as LP-HFY01, CQPC08, and TWK10, exhibited good free radical scavenging effect (11–14).

On the other hand, inflammatory bowel disease (IBD) refers to a chronic, progressive and recurrent intestinal disease characterized by chronic inflammation in the intestinal mucosa, and mainly includes ulcerative colitis (UC) and Crohn's disease (CD) (15–17). The current treatment of IBD mainly depends on long-term conventional anti-inflammatory treatment, which usually leads to drug intolerance or intolerance and limits the quality of life of patients (18). The pathogenesis of IBD is complex and diverse, resulting from the combined action of various factors, such as genetic factors, environmental factors, intestinal microbiota changes, and oxidative damage, immune dysfunction and psychological factors (19, 20). However, oxidative stress plays a key role in the development of intestinal injury in IBD, because it is primarily involved in the abnormal immune and inflammatory response (18). During the active disease phase, activated white blood cells will not only generate a wide spectrum of pro-inflammatory cytokines, but also excess oxidative reactions (18). It also has been proved that the improvement of antioxidant capacity by antioxidant active substances can effectively reduce the inflammatory damage caused by IBD (16).

Considering the antioxidative effect of fermented soymilk, we expect the fermented soymilk to has a preventive effect on UC. However, identifying whether the soymilk fermented by commercial *Lactobacillus plantarum* and other lactic acid bacteria (LAB) can suppress the oxidative stress, and maintain the redox homeostasis of the body, as well as confirming if we can improve the antioxidative effect by strain selection and changing preparation processing, still need further efforts. And very few studies have focused on the anti-inflammatory effect of soymilk *via* lactic acid bacteria fermentation.

Thus, this paper mainly discussed the effects of soymilk preparation process, different commercial LAB, and fermentation time on the antioxidant activity of fermented soymilk, and studied the preventive effects of soymilk fermented by commercial LAB on dextran sodium sulfate (DSS)-induced colitis in mice. This work provides a theoretical basis for the therapeutic effect of fermented soymilk and a reference for the preparation of fermented soymilk with higher nutritional value.

## 2. Materials and methods

### 2.1. Experimental reagents and strains

Caco-2 cells were purchased from the Shanghai Cell Bank of Chinese Academy of Sciences. The strains, including L571 (*Lactobacillus plantarum*); YF-L904, YF-L903, and Mild1.0 (mixed strains of *Streptococcus thermophilus* and *Lactobacillus Bulgarian*); and ST-M5 (*Streptococcus thermophilus*), were obtained from Chr. Hansen (Hoersholm, Denmark). DMEM high glucose cell culture medium and fetal bovine serum (FBS) were purchased from Corning Technology Co., LTD. The iron reduction capacity test kit, ABTS free radical scavenging capacity test kit, and PBS buffer were purchased from Solarbio Technology Co., LTD. MTT was purchased from Sigma-Aldrich. The reactive oxygen species (ROS) detection kit, lipid oxidation (MDA) detection kit, total SOD activity detection kit (WST-8 method), ELISA kits of interleukin-6 (IL-6), interleukin-1β (IL-1β), and tumor necrosis factor-α (TNF-α) were purchased from Beyotime Biotechnology Co., LTD. T-AOC, T-SOD, and MDA commercial kits were purchased from Nanjing Jiancheng Bio-engineering Insititute.

### 2.2. Preparation of soymilk and fermented soymilk

#### 2.2.1. Soymilk preparation using conventional method

Soymilk was prepared similarly to our previous reports (21, 22). Briefly, the selected soybeans (100 g) were washed and immersed in double-distilled water for 12 h at 4°C. Then the soaked soybeans were drained and ground in a soymilk machine with double-distilled water (700 mL) at room temperature. The obtained homogenate was filtered by a defatted cotton sheet to eliminate insoluble residues, and the filtrate was heated in boiling water bath for 5 min at 95°C. Subsequently, the soymilk was cooled to room temperature immediately by ice water bath. Soymilk prepared by this conventional method was coded as CN-20.

#### 2.2.2. Soymilk preparation using hot-water blanching method

**Scheme 1:**
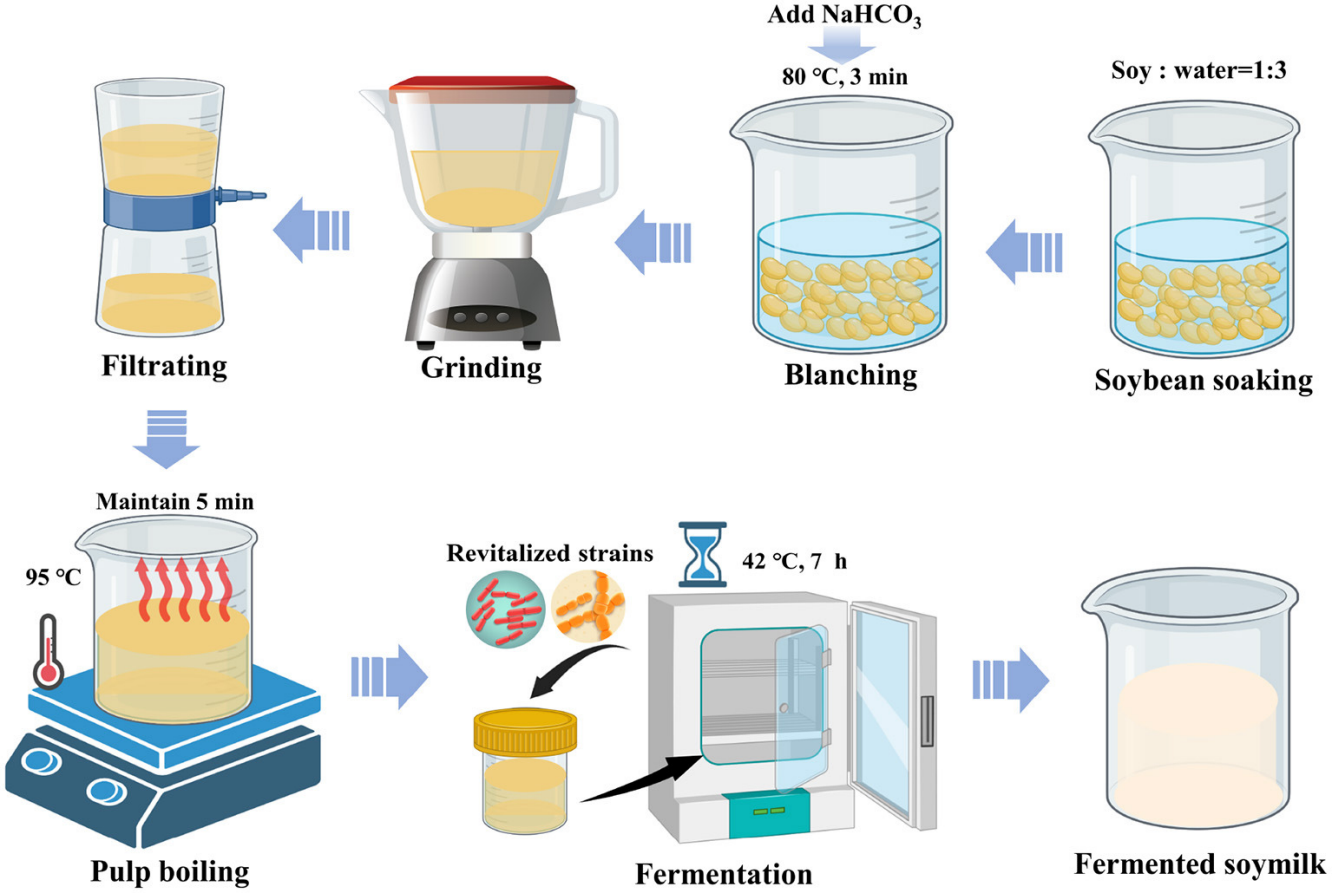
Fermented soymilk preparation process.

Another set of soymilk samples were prepared using a hot-water blanching approach as reported in our pervious works (22, 23). Briefly, as shown in Scheme 1, a total of 100 g soybeans were washed and immersed in double-distilled water for 12 h at 4°C. The soaked soybeans were then drained and blanched using NaHCO_3_ solution (0.04 mol/L, 300 mL) for 3 min at 80°C to deactivate lipoxygenases. Subsequently, the soybeans and blanching water were poured in a grinder contained with 400 mL of hot double-distilled water (80°C), and ground for 3 min. The obtained homogenate was filtered by a defatted cotton sheet to eliminate insoluble residues, and the obtained raw soymilk was heated in boiling water bath for 5 min at 95°C. After that, the soymilk was immediately cooled to room temperature by ice water bath. The corresponding soymilk prepared with the blanching treatment was coded as BT-80.

#### 2.2.3. Preparation of fermented soymilk

Fermented soymilk was prepared according to pervious reports with some modifications (24). To obtain fermented soymilk, the lyophilized powders of L571, YF-L904, YF-L903, Mild1.0, and ST-M5 were added in 100 mL of soymilk at the ratio of 0.02% (w/v). The mixture was evenly stirred and then incubated at 42°C. In general, the fermentation was terminated when the titrated acidity reached 60 °T. Next, they were taken out and freeze dried into powder and stored in refrigerator at −18°C. The YF-L903 fermented soymilk powder was used for animal experiments. Considering that sucrose was generally added to improve the flavor and taste in actual factory fermentation, the sucrose (3%) here was also added during the preparation of fermented soymilk in animal experiments (fermented soymilk coded as FSM).

#### 2.2.4. Preparation of the methanol extracts of soymilk (SE) and fermented soymilk (FSE)

To investigate the antioxidant effect, the soymilk powder and fermented soymilk powder were extracted with 0–100% methanol by stirring at 25°C for 12 h and filtered through filter paper. The methanol in the filtrate was removed by rotary evaporation, and the remaining part was freeze-dried into powder (soymilk extract coded as SE and fermented soymilk extract coded as FSE), which was collected and stored in the refrigerator at −18°C before using in antioxidative experiments.

### 2.3. Determination of nutritional contents

#### 2.3.1. Determination of total phenolic content in methanol extracts of soymilk and fermented soymilk

The total phenolic content was determined by Folin-Ciocalteu method with minor modifications (14). In detail, 100 μL of the extracts was added in the mixture of 2.95 mL of distilled water, 250 μL of Folin-Ciocalteu phenol reagent reagent, and 750 μL of NaCO_3_ (7%). After 8 min reaction, 950 μL of distilled water was added. Then the reaction continued for 2 h, protected from light at room temperature. After that, the absorbance at 765 nm was measured. Standard curves were obtained using gallic acid as a standard sample. The measurement was repeated three times for each sample. The total phenolic content was expressed as gallic acid equivalents per gram of sample (mg GAE/g).

#### 2.3.2. Determination of flavonoid content in methanol extracts of soymilk and fermented soymilk

The experiment was operated according to the instruction of plant flavonoid content detection kit (Beijing Solarbio Technology Co., LTD). In alkaline nitrite solution, flavonoids and aluminum ions form a red complex with characteristic absorption peak at 470 nm. The content of flavonoids in the extract can be calculated by measuring the absorbance at 470 nm. Standard curves were obtained using rutin as a standard sample. The measurement was repeated three times for each sample. The flavonoid content of the samples was expressed as rutin equivalents per gram of sample (mg GAE/g).

#### 2.3.3. Determination of isoflavones content in soymilk and fermented soymilk by HPLC

The isoflavone content was determined by high-performance liquid chromatography (HPLC) in AOAC (25). Isoflavones in the samples were determined qualitatively and quantitatively using Shimadzu (Japan) HPLC system. The analytical conditions were adopted as follow, Column: 15 × 4.0 mm C18, mobile phase A: water + methanol + glacial acetic acid (88 + 10 + 2), mobile phase B: methanol + glacial acetic acid (98 + 2), flow rate: l mL/min. Detection wavelength: 260 nm. Column temperature: 50°C. Injection volume: 20 μL. The analysis time was 40 min/sample.

### 2.4. *In vitro* evaluation of the antioxidant capacity of fermented soymilk

#### 2.4.1. DPPH radical scavenging ability

The 2,2-diphenyl-2-picrylhydrazyl (DPPH) radical scavenging activity was determined using the method of Yu et al. with some modifications (26). Briefly, a total of 0.1 mM DPPH solution was prepared with 95% ethanol and stored in the dark. Next, 0.2 mL of extract was mixed with 0.8 mL of DPPH solution, and the mixture was shaken vigorously to improve the uniformity. The obtained 1 mL samples were then incubated in the dark for 30 min, and the absorbance of the supernatant was measured at 517 nm (A_1_). The absorbance of 0.2 mL DPPH solution prediluted with 0.8 mL ultrapure water was measured as blank control (A_0_). The absorbance of 0.2 mL test sample solution prediluted with 2 mL ethanol was used as standard control (A_2_). The DPPH radical scavenging rate was calculated according to the Formula (1).


(1) $$\begin{array}{l} DPPH\;radical\;scavenging\;rate\%\;=\;1-\frac{\left(A_{1}A_{2}\right)}{A_{0}}\times 100\% \end{array}$$

#### 2.4.2. Determination of ABTS radical scavenging ability

This experiment was operated according to the instructions of ABTS radical scavenging ability test kit. ABTS was oxidized to form stable blue-green cationic ABTS radicals with maximum absorption peaks at 405 nm or 734 nm. When ABTS radical solution was added with the sample, the antioxidant components could react with ABTS radical and make the reaction system fade, and the absorbance at 405 nm decreased. The change in absorbance at 405 nm was proportional to the degree of free radical removal in a certain range. The decrease in absorbance was measured to evaluate the ABTS free radical scavenging ability of the sample. A_1_ denotes the absorbance of samples, A_0_ denotes the absorbance of blank control, and A_2_ denotes the absorbance of standard control. ABTS radical scavenging rate was calculated according to the Formula (2).


(2) $$\begin{array}{l} ABTS\;radical\;scavenging\;rate\%\;=\;\text{1}-\frac{\left(A_{1}A_{2}\right)}{A_{0}}\times 100\% \end{array}$$

#### 2.4.3. Determination of metal ion-chelating ability

The metal ion-chelating activity was measured by following the previous report with minor modifications (27). A total of 2 mL of sample solution was obtained by mixing 0.1 mL of 2 mM FeCl^2^ with 3.9 mL of distilled water in a reaction tube. Subsequently, ferrozine solution (0.1 mL of 5 mM) was then added and mixed vigorously. The mixture was set aside for 10 min at room temperature, and then photo-absorbance was detected at 562 nm. Distilled water without added sample was used as the blank. A_1_ denotes the absorbance of samples, A_0_ denotes the absorbance of blank control and A_2_ denotes the absorbance of standard control. Metal ion-chelating rate was calculated according to the Formula (3).


(3) $$\begin{array}{l} Metal-ion\;chelating\;rate\;\%\;=\;1-\frac{\left(A_{1}A_{2}\right)}{A_{0}}\times 100\% \end{array}$$

#### 2.4.4. Determination of total reduction capacity

The experiment was operated according to the instructions of the total antioxidant capacity assay kit (Beijing Solarbio Technology Co., LTD.). In acidic environment, the antioxidant components of the tested samples can reduce Fe^3+^-tripyridinium triazine (Fe^3+^-TPTZ) to produce blue Fe^2+^-TPTZ, which has a maximum absorption at 593 nm. The enhanced degree of absorbance at 593 nm indicates the antioxidant capacity of the sample. In our experiments, standard curve was collected from FeSO_4_•7H_2_O, and the total reduction capacity was expressed as the content of Fe^2+^ in the solution.

### 2.5. Cell experiments

#### 2.5.1. Caco-2 cell culture

The Caco-2 cells were cultured in 10 mL DMEM medium (containing 10% FBS, 100 U/mL penicillin, and 0.1 mg/mL streptomycin) at 37°C in a fully humidified atmosphere containing 5% CO_2_. The medium was renewed every 48 h and cells were subcultured using 0.25% trypsin-EDTA when they reached 80–90% confluency.

#### 2.5.2. H_2_O_2_-induced oxidative damage model in Caco-2 cells

First, the Caco-2 cells (100 μL, 5 × 10^4^ cells/mL) were seeded in the wells of 96-well plate. After incubation for 18 h at 37°C, the grown medium was taken out, and the wells were washed with PBS twice. Then, the cells were treated with 100 μL of DMEM medium of H_2_O_2_ (0.25–8 mM), and the control group was added with 100 μL of DMEM medium without H_2_O_2_, and both incubated for 1–3 h. At last, the culture medium was removed by a pipette and the cells were washed twice with PBS. The cell viability was determined by MTT method same as described above.

#### 2.5.3. Determination of cytotoxicity

The cytotoxicity of the samples was determined by MTT [3-(4, 5-dimethylthiazole-2-yl)-2, 5-diphenyltetrazolium bromide]. The Caco-2 cells (100 μL, 5 × 10^4^ cells/mL) were seeded in the wells of 96-well plate. After incubating for 18 h at 37°C. Thereafter, the culture medium was changed to one that contained the sample to be tested, and the culture was continued for 24 h. 20 μL MTT (0.5 mg/mL) was added for further incubation for 4 h. Then, the culture medium was removed, 150 μL dimethyl sulfoxide (DMSO) was added, and the absorbance was measured at 490 nm after shaking slowly for 10 min to completely dissolve the purple color at the bottom completely. The results were expressed as the cell viability (%) of the experimental group compared with the control group.

#### 2.5.4. Determination of the cell viability of H_2_O_2_-induced oxidative damage

Caco-2 cells (100μL,5 × 10^4^ cells/mL) were seeded in the wells of a 96-well plate. After incubating for 18 h at 37°C, the grown medium was taken out, and the wells were washed with PBS twice. Subsequently, the cells were treated with soymilk or fermented soymilk at the concentration of 125, 250, 500, and 1,000 μg/ml. After that, the culture medium was removed and washed with PBS for two to three times, the medium without H_2_O_2_ was added to the control group, whereas the medium containing H_2_O_2_ was added to the model and experimental groups to make the final H_2_O_2_ concentration reach 1 mM, and the incubation was continued for 1 h. After removing the culture medium, the cells were washed twice with PBS, and the cell viability was determined by MTT method.

#### 2.5.5. Determination of ROS level in Caco-2 cells by H_2_O_2_-induced oxidative damage

Intracellular ROS levels were measured using reactive oxygen species assay kit. Caco-2 cells (100 μL, 5 × 10^4^ cells/mL) were seeded in the wells of 96-well plate and incubation according to the method mentioned above. After the culture medium was removed and washed twice with PBS, 100 μL serum-free complete medium containing 10 μM DCFH-DA probe was added and cultured for 20 min. Cells were washed twice with serum-free complete medium, and their fluorescence intensity was measured using a microplate reader at 488 nm excitation wavelength and 525 nm emission wavelength.

#### 2.5.6. Determination of MDA level in Caco-2 cells by H_2_O_2_-induced oxidative damage

The intracellular MDA levels were measured using lipid peroxidation MDA assay kit. First, the cells were treated according to the aforementioned method. Then the culture medium was removed and washed twice with PBS. RIPA cell lysate was added to lyse cells, and the protein concentration of samples in each group was determined by using the BCA protein concentration assay kit. A total of 0.37% TBA storage solution and MDA test solution were prepared. The samples/standards and MDA test solution were added and mixed according to the instructions, heated at 100°C for 15 min, cooled to room temperature, and centrifuged for 10 min (1,000 × g). The absorbance of the supernatant was measured at 532 nm, and the molar concentration of MDA was calculated according to the standard curve. The results were expressed as the content of MDA per unit weight of protein (μmol/mg protein).

#### 2.5.7. Determination of SOD activity in Caco-2 cells by H_2_O_2_-induced oxidative damage

Total SOD assay kit with WST-8 was used in this experiment. After following a commonly used incubation process mentioned above, the culture medium was removed and washed twice with PBS, and the SOD sample preparation solution was added to prepare the cell lysate. WST-8/enzyme working solution was prepared by mixing SOD detection buffer solution, WST-8 and enzyme solution in a proportion. The reaction starting solution (40 × ) was diluted to produce the reaction starting working solution. In turn, the samples to be tested, SOD detection buffer solution, WST-8/enzyme working solution, and reaction starting solution were added according to the instructions, and the reaction was carried out at 37°C (30 min). The absorbance of the samples was measured at 450 nm, and SOD activity was expressed as U/mg protein.

### 2.6. Animal experiments

#### 2.6.1. Animals and experimental design

C57BL/6JNifdc mice (male, 6–8 weeks old) were obtained from Vital River Laboratory Animal Technology Co., Ltd. (Beijing, China) and kept in polypropylene cages at 25 ± 2°C, under standard conditions of 12-h light/dark cycle with a relative humidity of 55 ± 5%. All experimental procedures were approved by the Animal Ethics Committee of Pony Testing International Group Co., Ltd (Approval No.: PONY-2022-FL-12). Mice had free access to basal diet (Vital River Laboratory Animal Technology Co., Ltd., Beijing, China) and water. After one-week adaption, mice were randomly divided into four groups (*n* = 8 in each group): (1) normal control group (Control), (2) DSS-induced colitis model group (DSS), (3) low-dose fermented soymilk group (DSS+L-FSM), and (4) high-dose fermented soymilk group (DSS+H-FSM).

**Scheme 2:**
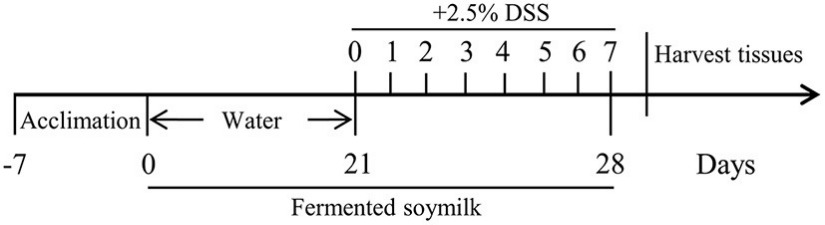
Diagram illustrating the protocol of mice model of colitis.

The procedures were conducted as follows: During the whole experimental period, Group 1 (Control) and Group 2 (DSS) were given 0.2 mL of 0.9% normal saline every day by gavage. Meanwhile, mice in Group 3 (DSS+L-FSM) and Group 4 (DSS+H-FSM) were gavaged with 0.2 mL FSM (0.8 g/kg BW) and FSM (1.6 g/kg BW), respectively. From day 21 of the experimental period, nearly all mice, except normal control mice, were changed to free drinking deionized water containing 2.5% DSS (purchased from MP biomedicals, USA) as shown in Scheme 2.

#### 2.6.2. Assessment of colitis

Body weight was measured daily for the entire duration of the study. Disease activity index (DAI) was evaluated to assess the severity of the colitis by combining scores of body weight loss, diarrhea of the stool, and the extent of the blood in the feces (28). From day 21 to day 28, body weight and DAI was measured daily. At day 28, all mice were sacrificed by cervical dislocation. The colon was collected and flushed by ice-cold PBS, and then the length was recorded. A section of the colon (about 1 cm) between the one third of the length to the midpoint was fixed in 10% formalin and embedded in paraffin. After being sectioned at about 5 μm, it was stained with hematoxylin and eosin (H&E). The histological analysis of stained colon section was conducted by optical microscopy and scored based on a scale that graded the extent of Mucous membrane (0–5), Destruction of epithelium and glands (0–3), Enlargement of gland recess (0–3), Reduction of goblet cells (0–3), Inflammatory cell infiltration (0–3), edema (0–3), Mucosal hemorrhage (0–3), Crypt abscess (0–3) and Dysplasia (0–3), respectively (29, 30).

#### 2.6.3. Inflammatory cytokines and antioxidant indexes assay

At day 28, blood was obtained from the eyeballs of the mice, and centrifuged by a high-speed freezing centrifuge at 3,000 r/min for 10 min. The serum was then stored at an ultra-low temperature refrigerator (−80°C). ELISA kit (Beyotime Biotechnology Co., LTD.) was used to analyze the levels of TNF-α, IL-6, and IL-1β in the serum according to the instruction, while antioxidant indexes such as T-AOC, T-SOD, and MDA were quantified using commercial kits (Nanjing Jiancheng Bio-engineering Insititute, Nanjing, China).

### 2.7. Statistical analysis

GraphPad Prism 9.0 and Spass 17.0 software were used to calculate and analyze the results, and which are presented as the mean±standard deviation ($\overset{\bar{}}{X}\;\pm$ SD). The differences among the datasets were compared using one-way ANOVE followed by Tukey's HSD multiple comparison *post-hoc* tests. When *p* < 0.05 indicates statistical significance.

## 3. Results

### 3.1. Effect of fermentation conditions on antioxidant activity of soymilk *in vitro*

**Figure 1:**
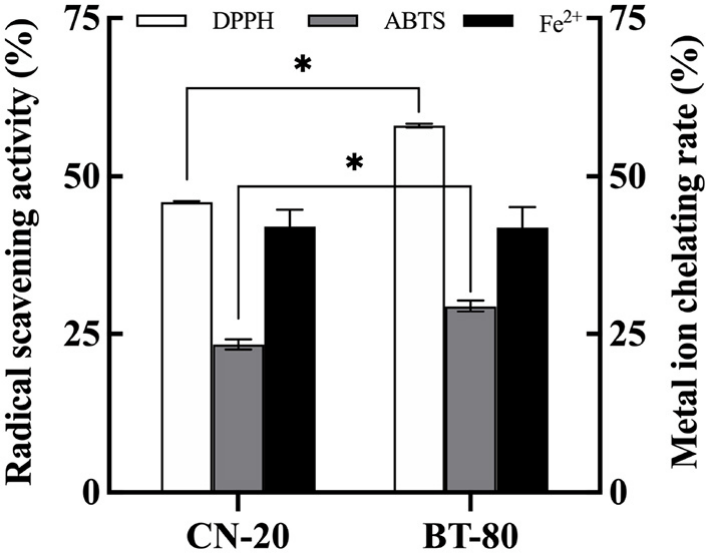
Effects of soymilk production processes on the antioxidant properties of fermented soymilk. * indicates *p* < 0.05 according to Tukey's HSD tests.

In order to obtain fermented soymilk with high antioxidative activity, the effect of soymilk preparation process on the *in vitro* antioxidant activity of fermented soymilk was investigated as shown in Figure 1. The BT-80 method is commonly used process in the preparation of soymilk drinks to remove the bean smell (24). While the CN-20 method is a traditional process for soymilk preparation. The DPPH and ABTS radical scavenging abilities of the BT-80 groups were 58.63, and 30.13%, respectively, which were significantly higher than those of the CN-20 groups (45.81, and 23.94%, respectively). Meanwhile the Fe^2+^ chelation rates were all ~41%. This result showed that the processing technology of fermented soymilk would influence its free radical scavenging capacity to some extent. The BT-80 method can improve the antioxidant capacity possibly due to the inactivation of enzymes, the existence of antioxidant physiological active substances and the accessibility of LAB fermentation. Consequently, the BT-80 method was chosen to prepare the soymilk in the following experiments.

To screen out an excellent commercial LAB product, the *in vitro* antioxidant activity of fermented soymilk prepared by five kinds of LAB (L571, Mild1.0, ST-M5, YF-L903, and YF-L904) were determined and compared as shown in Table 1, including the DPPH radical scavenging ability, ABTS radical scavenging ability, and Fe^2+^ chelation ability. The ABTS and DPPH radicals scavenging activity in the SM group (unfermented) were 14.96 and 39.37%, respectively, and the Fe^2+^ chelation rate was 50.36%. After fermentation, the ABTS and DPPH radicals scavenging ability were significantly enhanced regardless of the LAB strain used in fermentation. The Fe^2+^ chelation rate was also significantly increased, except for L571 fermented soymilk. This result further shows that lactic acid bacteria fermentation can improve the free radical scavenging and antioxidant activity of soybean milk. However, the extent of improving the free radical scavenging capacity differed among the LAB. The ABTS radical scavenging ability and the Fe^2+^ chelation rate of group YF-L903 was significantly higher than that of group L571. These results indicated that the free radical scavenging ability and Fe^2+^ chelation rate of fermented soymilk are closely related to the fermentation strain (31, 32). Take the three indicators into consideration, the YF-L903 would be the best choice for us.

**Table 1:** Antioxidant activity of soymilk fermented by different commercial lactic acid bacteria.

**FSM with different lactic acid bacteria**	**ABTS scavenging activity (%)**	**DPPH scavenging activity (%)**	**Fe^2+^chelation rate (%)**
SM	14.96 ± 0.83^a^^A^	39.37 ± 0.04^a^	50.36 ± 0.03^b^
L571	16.76 ± 0.64^a^	42.32 ± 0.02^a^	32.10 ± 0.67^a^
Mild1.0	23.55 ± 2.38^b^	43.11 ± 1.43^a^	66.26 ± 0.27^d^
ST-M5	25.14 ± 0.41^bc^	44.09 ± 0.64^a^	68.10 ± 0.15^d^
YF-L904	28.37 ± 0.62^bc^	43.04 ± 2.23^a^	63.49 ± 0.29^c^
YF-L903	30.09 ± 0.51^c^	43.80 ± 0.14^a^	63.99 ± 0.23^c^

A Mean value ± standard deviation.

a−d Mean values with different letters in the same column are significantly different (*p* < 0.05), per Tukey's HSD test.

SM was the soymilk group without strain; L571, YF-L904, YF-L903, Mild1.0, and ST-M5 were the commercial fermentation strains, in which L571 was Lactobacillus plantarum, ST-M5 were Streptococcus thermophilus, YF-L904, YF-L903, and Mild1.0 were mixed strains of Streptococcus thermophilus and Lactobacillus germanicus.

It is well known that soybean isoflavones exhibit weak polarity, are soluble in 80% methanol or ethanol, and have various activities, such as preventing obesity, lowering blood sugar levels, reducing the risk of osteoporosis and breast cancer, and showing antioxidant and anti-inflammatory effects (33, 34). Soybean isoflavones are mainly composed of aglycone isoflavones (daidzein, genistein, glycitein) and glycoside isoflavones (daidzin, genistin, glycitin). In the fermentation process of soymilk, β-glucosidase in microorganisms can convert glycosidic flavonoid into aglycone flavonoid (35), so that the antioxidant and anti-inflammatory function of fermented soymilk is significantly improved (36).

**Figure 2:**
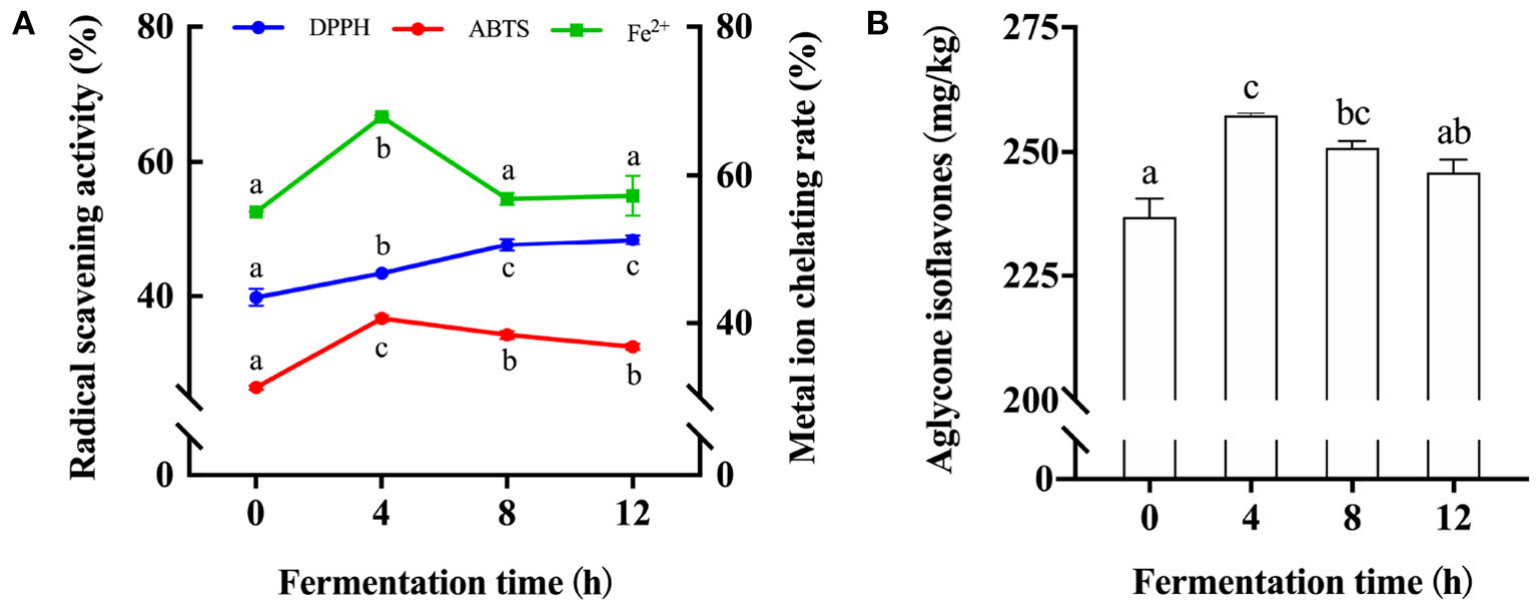
Changes of antioxidant activity and Aglycone isoflavone content in soymilk at different fermentation times. **(A)** Antioxidant activity of soymilk at different fermented times; **(B)** Aglycone isoflavone content in soymilk at different fermentation times; (a–c) Mean values with different letters in the same column are significantly different (*p* < 0.05), per Tukey's HSD test.

Thus, we then investigated the effect of fermentation time on the antioxidation ability and the content of aglycone isoflavones in fermented soymilk. The DPPH and ABTS radical scavenging activity and metal ion chelating rate in soymilk and fermented soymilk were determined as shown in Figure 2A, in which the values for unfermented soymilk were 38, 26, and 55%, and they were increased to 44, 37, and 68% after 4 h of fermentation. However, the ABTS radical scavenging activity and metal ion chelating rate were decreased to 37, and 68% after 12 h of fermentation. These results showed that the antioxidant activity in soymilk increased first and then decreased with the prolongation of fermentation time. Besides, as shown in Figure 2B, the content of aglycone isoflavones in soymilk was 235 mg/kg, which increased to 257 mg/kg after 4 h of fermentation. However, this amount decreased slightly and finally became stable with continuous fermentation. Therefore, fermentation time has a significant influence on the content of active components in fermented soymilk and results in the difference in the antioxidative effects. The antioxidant activity of fermented soymilk is directly proportional to its content of aglycone isoflavones (36), and is significantly affected by the total isoflavone content of soybean and the fermentation time (37). Therefore, it is suggested to optimize the fermentation time of soy milk in practical application to maximize the antioxidant capacity of fermented soymilk. The above results showed that fermentation strain, fermentation time and soybean milk preparation technology are the key factors affecting the antioxidant activity of fermented soymilk. And the BT-80 method, LAB YF-L903 LAB and 8h was adopted to prepare the fermented soymilk for the subsequent experiments.

### 3.2. *In vitro* antioxidative effects of soymilk and fermented soymilk

**Figure 3:**
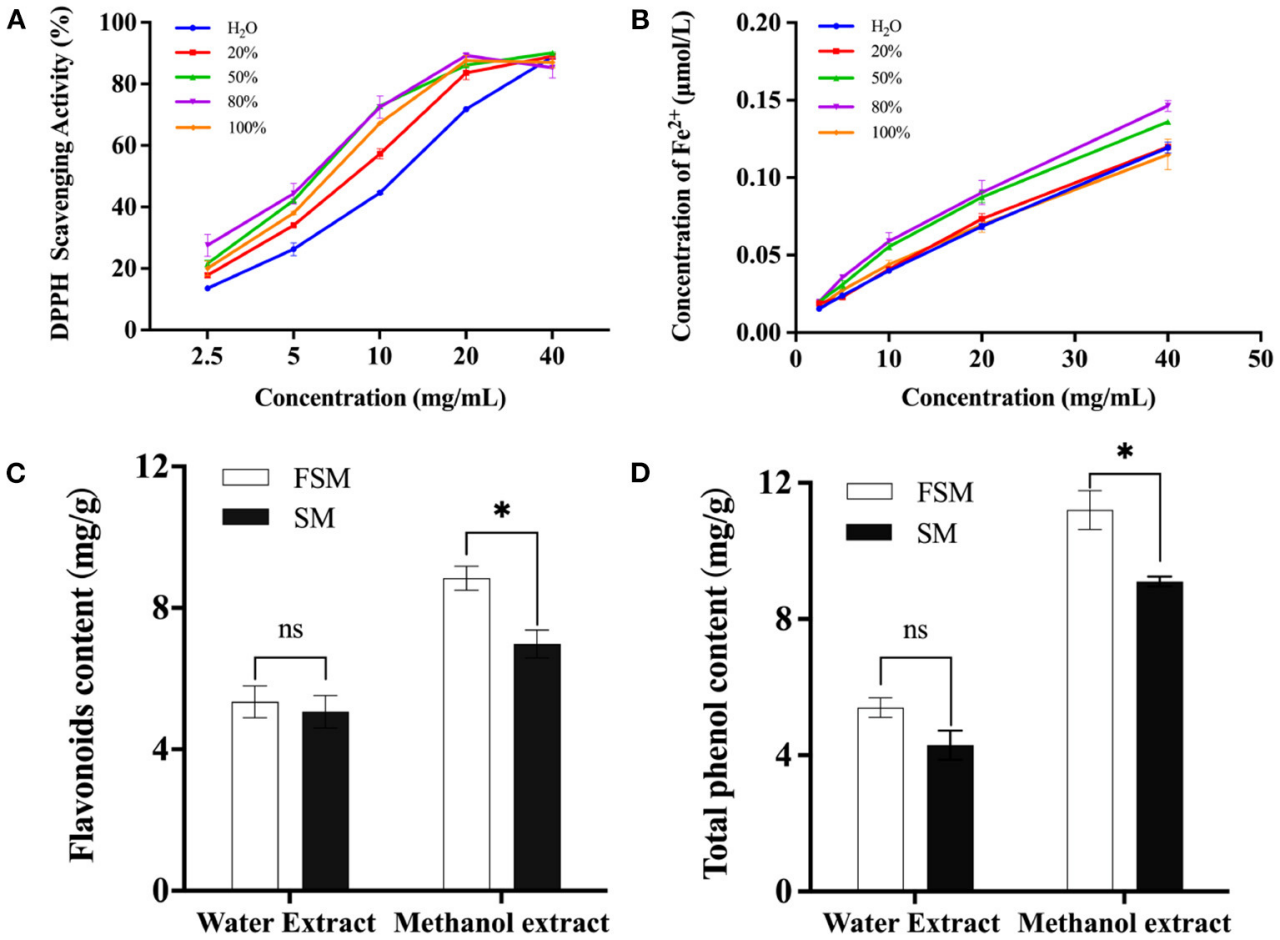
Effect of fermented soymilk active components extraction methods on antioxidant activity; **(A)** DPPH scavenging ability of fermented soymilk extract with different methanol concentrations; **(B)** Total reducing ability of the extract with different methanol concentrations; **(C)** Flavonoid content of the extract with water and 80% methanol; **(D)** Total phenolic content of the extract with water and 80% methanol. * indicates *p* < 0.05 according to Tukey's HSD tests.

Here, the antioxidant activities of solvent extracts with different polarity were analyzed. Figures 3A, B show that regardless of the polarity of the extractor, the free radical scavenging rate and total reducing capacity gradually increased with the increase of the extract concentration and decrease of the polarity of the extractor. The maximum free radical scavenging rate reached when the concentration of methanol was 80%. Therefore, 80% methanol extract can reflect the antioxidant activity of soymilk or fermented soymilk. Since isoflavones, saponins, and phenolic compounds in soybean have good antioxidant activities (9, 38), we also analyzed the flavonoid and total phenol contents in soymilk and fermented soymilk as shown in Figures 3C, D. The contents of flavonoids and total phenols in the 80% methanol extract were 6.96–8.53 mg/g and 9.01–10.08 mg/g, respectively, which were significantly higher than those in the water extract. In addition, the contents of flavonoids and phenolic compounds in the FSE were 8.53 and 10.08 mg/g, respectively, which were also significantly higher than those in the SE.

**Figure 4:**
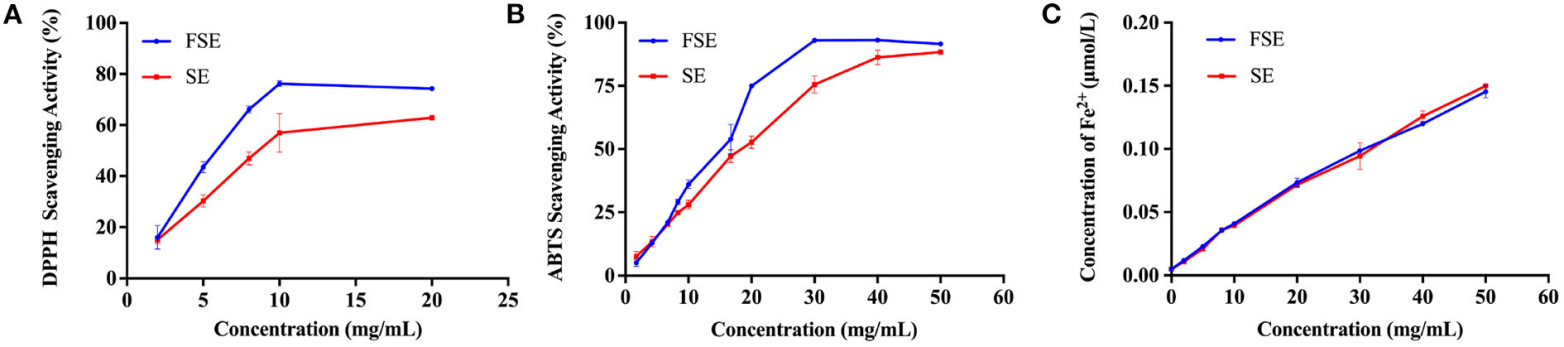
Antioxidant capacity of 80% methanol extracts of soymilk and fermented soymilk. **(A)** DPPH free radical scavenging ability; **(B)** Radical scavenging ability of ABTS; **(C)** Iron reduction capacity. FSE: 80% methanol extract of fermented soymilk, SE: 80% methanol extract of soymilk.

The antioxidative effects of the SE and FSE were further analyzed based on DPPH radical scavenging ability, ABTS radical scavenging ability, and iron reduction ability. As shown in Figure 4, the DPPH radical scavenging ability increased with the extract concentration and reached stability at 10 mg/mL. Noteworthy is that the DPPH radical scavenging ability of fermented soymilk was always 25% higher than that of soymilk (Figure 4A). The ABTS radical scavenging ability exhibited a similar effect to that of DPPH, and the maximum value of fermented soymilk was 93.2% at 30 mg/mL, obvious higher than the 82% of soymilk (Figure 4B). The iron reducing ability of the extracts increased linearly with the increase of extract concentration, but there was no significant difference was observed between soymilk and fermented soymilk. A similar phenomenon was also reported by Lee and Chen et al., who showed that *Lactobacillus plantarum* P1201 or *Lactococcus acidophilus* MF204 fermented soymilk had stronger DPPH and ABTS radical scavenging ability than soymilk possibly due to the significant increase in conjugated linoleic acid (CLA) content and the conversion of glycosidic isoflavones to more physiologically active aglycone isoflavones during fermentation (39, 40).

**Figure 5:**
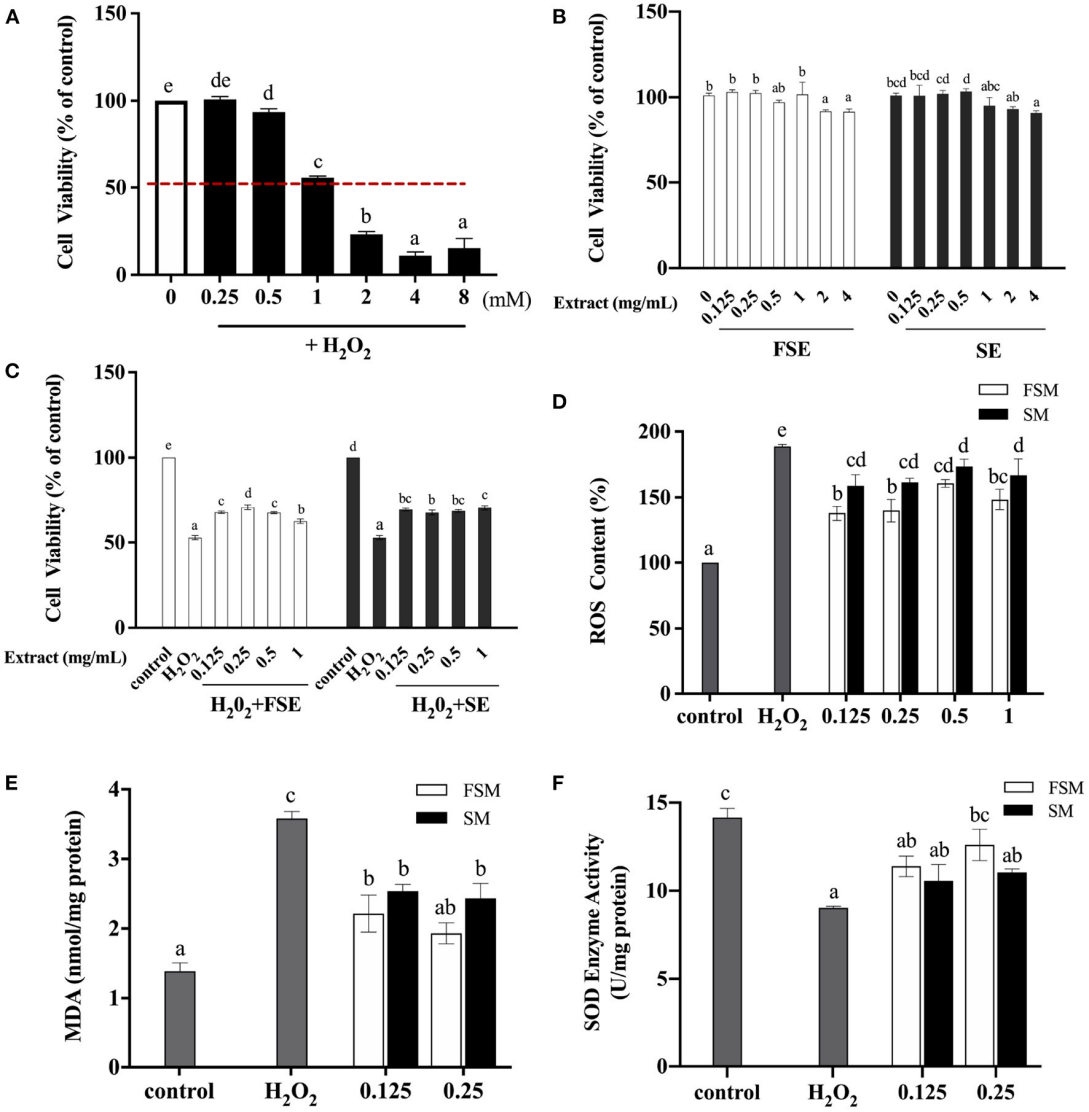
Protective effect of fermented soymilk on H_2_O_2_-induced oxidative damage in Caco-2 cells. **(A)** Effect of different concentrations of H_2_O_2_ on the activity of Caco-2 cells; **(B)** Effects of different concentrations of FSE and SE on the activity of Caco-2 cells; **(C)** Effects of different concentrations of FSE and SE on the activity of H_2_O_2_-induced Caco-2 cells; **(D)** Effects of FSE and SE on the content of reactive oxygen species (ROS) in H_2_O_2_-stimulated Caco-2 cells; **(E)** Effects of FSE and SE on the content of malondialdehyde (MDA) in H_2_O_2_-stimulated Caco-2 cells; **(F)** Effects of FSE and SE on the content of superoxide dismutase (SOD) in H_2_O_2_-stimulated Caco-2 cells. (a–d) Different letters indicate significant differences between groups according to Tukey's HSD test (*p* < 0.05).

The protective effects of soymilk and fermented soymilk against H_2_O_2_-induced oxidative stress in Caco-2 cells then were explored. As depicted in Figure 5, Firstly, the appropriate H_2_O_2_ concentration for induction was detected when the cells were in the half-life period. As shown in Figure 5A, the Caco-2 cell viability gradually decreased with the increase of H_2_O_2_ concentration. When the concentration of H_2_O_2_ reached 1 mM, the survival rate of Caco-2 cells decreased to about 50%. Thus, 1 mM H_2_O_2_-induced Caco-2 cells were chosen for the analysis of the protective effects of FSE and SE against oxidative stress. Cell viability, ROS release, and malondialdehyde (MDA) and superoxide dismutase (SOD) activity were measured.

The cytotoxic effect of FSE and SE was tested as shown in Figure 5B. The cell viability of the control group was 100%. When the concentrations of FSE and SE were 0.125, 0.25, 0.5, and 1 mg/mL, there was no significant difference in cell viability compared with the control group, indicating that such concentrations had no toxic effect on these cells. However, when the concentration of FSE and SE reached 2 and 4 mg/mL, the cell viability was decreased to about 90%, indicating a significant inhibitory effect on cell viability. Therefore, in subsequent experiments, the maximum concentration of samples was selected as 1 mg/mL.

Figure 5C shows that compared with the control group, the cell viability of the injury model group (H_2_O_2_) was reduced to 52.47%. When the concentration of SE and FSE was 0.125 mg/mL, the cell viability of the treatment group (SE+H_2_O_2_ and FSE+H_2_O_2_) was about 70%, which was significantly increased compared with that of the injury model group. This finding indicated that SE and FSE could help cells to resist the oxidative damage induced by H_2_O_2_. However, no significant increase in cell viability was observed with further increase of SE and FSE concentrations, indicating that their protective effect on injured cells was not dose-dependent.

H_2_O_2_ induction leads to excessive ROS production, which can drive DNA damage and genetic instability and trigger oxidative stress-induced cell death (41). Figure 5D shows that compared with the injury model group (H_2_O_2_), the SE and FSE groups had significantly decreased ROS content, implying the ROS scavenging function of both extracts. The ROS scavenging ability of FSE was stronger than that of SE. Moreover, ROS can attack polyunsaturated fatty acids in biofilms, resulting in the peroxidation of lipids to form MDA. Figure 5E shows that compared with the control group, the injury model group had significantly increased MDA concentration. Meanwhile the SE and FSE significantly suppressed the increase of MDA content. Compared with SE, FSE demonstrated a stronger inhibiting ability for MDA, indicating its enhanced oxidative damage protection ability.

The effects of FSE and SE pretreatment on the SOD activity in oxidation-damaged cells were investigated as shown in Figure 5F. SOD is an important antioxidant enzyme in living organisms and can scavenge superoxide anion radical produced in the body, thus protecting cells from oxidative damage. It is also an important factor in maintaining the balance between oxidation and anti-oxidation, thus reflecting the antioxidant capacity of the body. Compared with that in the control group, the SOD activity in cells was significantly decreased in the H_2_O_2_ group. Compared with that in the H_2_O_2_ group, the SOD activity significantly increased in FSE and SE groups. These results indicated that FSE and SE could suppress the increase of intracellular ROS content induced by H_2_O_2_, reduce the oxidative damage, and restore cell viability by enhancing SOD activity. Furthermore, fermentation could improve the protective effect of soymilk against H_2_O_2_-induced oxidative damage in Caco-2 cells. Qian et al. (14) also found that *Lactobacillus casei* 16 fermented soymilk has a protective effect against the oxidative damage in HepG2 cells, which may be related to the increase in the content of total phenols and free amino acids. Fermented soymilk can also remarkably enhance the antioxidant capacity of prematurely aging mice and hyperlipidemic rats (11, 12, 40).

### 3.3. Preventive effect of fermented soymilk on DSS-induced colitis in mice

**Figure 6:**
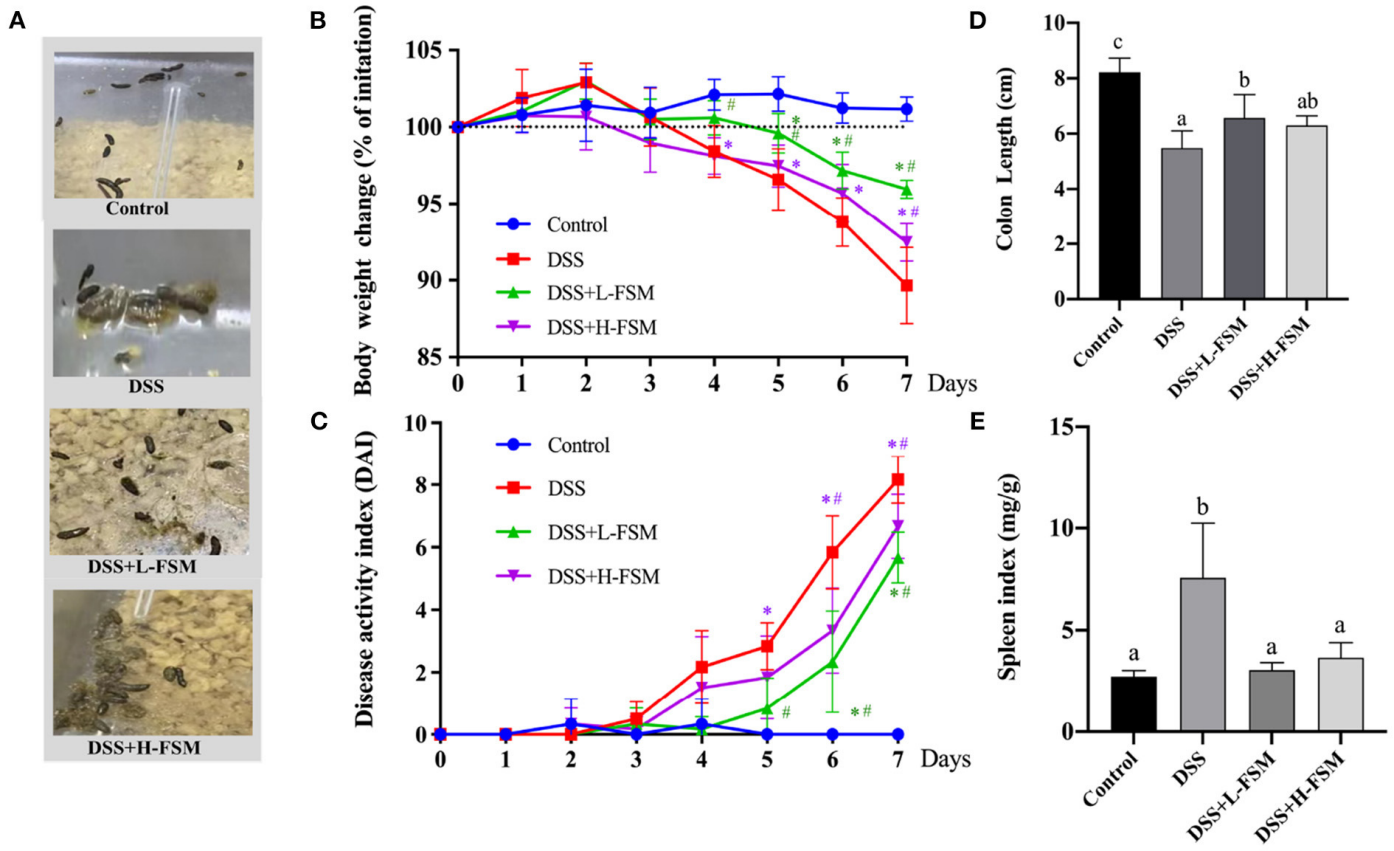
Effect of fermented soymilk on the health condition of DSS-induced colitis mice. **(A)** Fecal condition; **(B)** Daily body weight changes throughout the entire duration of the DSS treatment (*n* = 6 per group); **(C)** Disease activity index throughout the entire duration of the DSS treatment (*n* = 6 per group); **(D)** the lengths of colon (*n* = 6 per group); **(E)** Spleen-to-body weight ratio (*n* = 6 per group). Control, control group; DSS, colitis model group; DSS+L-FSM, low-dose fermented soymilk (0.8 g/kg BW) group; and DSS+H-FSM, high-dose fermented soymilk (1.6 g/kg BW) group. (a–d) Different letters indicate significant differences between groups according to Tukey's HSD test, *p < 0.05 relative to Control group, #*p* < 0.05 relative to DSS group.

In IBD, oxidative stress refers to an imbalance between the production and elimination of ROS, not only occurs in the inflamed intestinal mucosa but also extends into the deeper layers of the intestinal wall and is mirrored within the systemic circulation (42, 43). Considering the ROS scavenging ability of fermented soymilk, as well as its effect of maintaining the redox homeostasis in the cells, we further explored the prevention effect of fermented soymilk on colitis in mice. The experimental colitis was induced in mice by administering 2.5% DSS in water continuously for 7 days (Scheme 2). Physiological indices including fecal condition, weight changes, disease activity index (DAI), the length of colon and spleen-to-body weight ratio were recorded as shown in Figure 6 to investigate the prevention effect of fermented soymilk on DSS-induced colitis in mice. The mice in the control group had normal feces and colon length, no weight loss and DAI score, and a spleen-to-body weight ratio of 0.25%. After treatment with DSS, the mice showed loose stool. Compared with the DSS group, the group gavaged with fermented soymilk showed significantly improved fecal status and well-formed feces, regardless of low or high fermented soymilk dose (Figure 6A). In the DSS group, the body weight, and the colon length reduced significantly, DAI, and spleen-to-body weight ratio of mice increased visible. Compared with the DSS group, fermented soymilk intervention remarkably increased the body weight and colon length, and decreased the DAI in mice with colitis (Figures 6B–D). In particular, the spleen-to-body ratio was the same as that in the control group without significant difference (Figure 6E). The above experimental results directly demonstrated that lactic acid-fermented soymilk can enhance intestinal health and prevent colitis.

**Figure 7:**
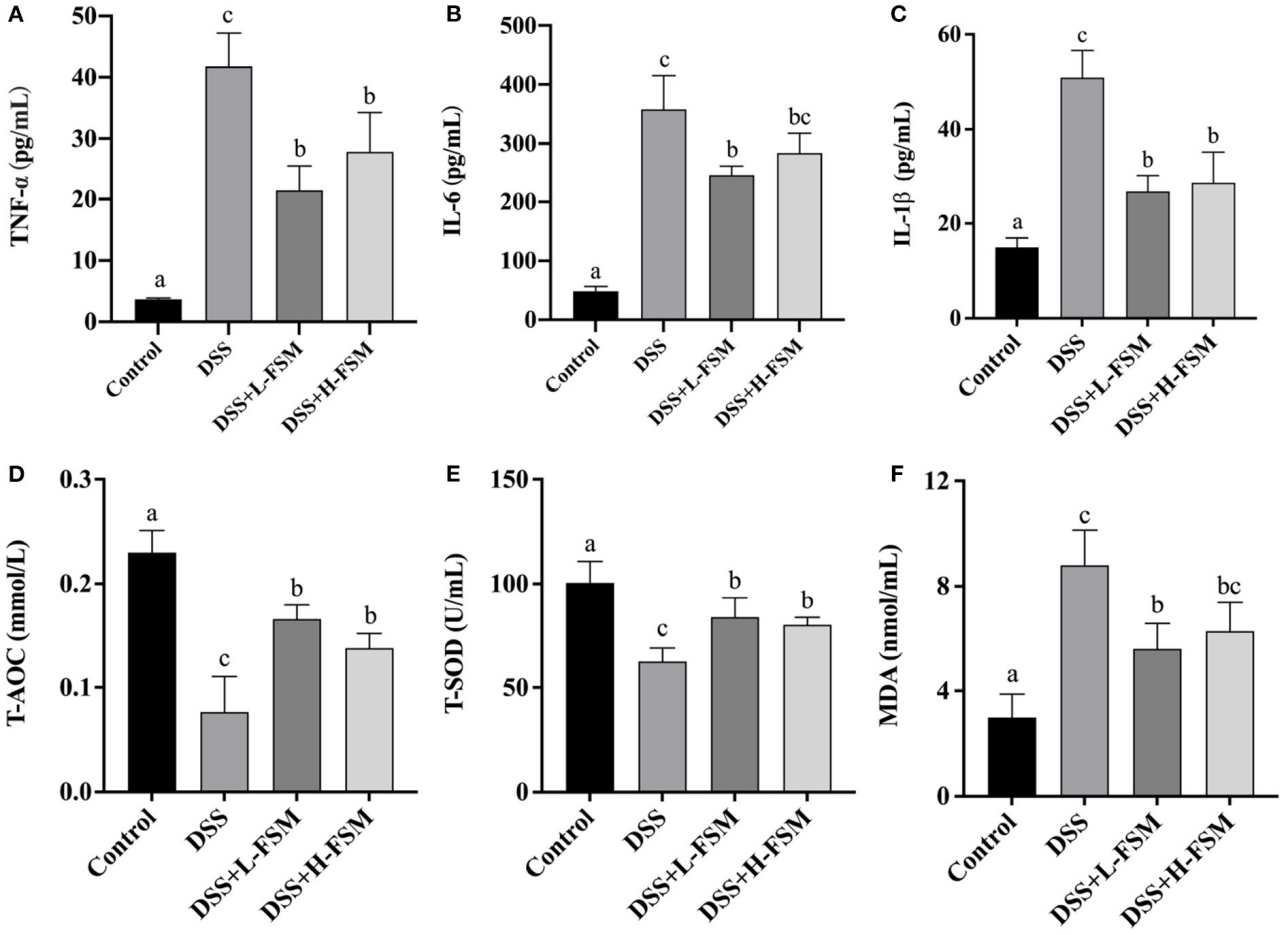
Effect of fermented soymilk on the release of blood inflammatory cytokines in mice with DSS-induced colitis. Concentrations of three pro-inflammatory cytokines, Tumor necrosis factor-α (TNF-α) **(A)**, interleukin-6 (IL-6) **(B)**, interleukin-1β (IL-1β) **(C)** in the serum (*n* = 6 per group). Concentrations of T-AOC **(D)**, T-SOD **(E)**, MDA **(F)** in the serum (*n* = 6 per group). (a–d) Different letters indicate significant differences between groups according to Tukey's HSD test (*p* < 0.05).

To further assess the impact of fermented soymilk on systemic response, pro-inflammatory cytokines in the serum was measured. TNF-α, IL-6, and IL-1β are inflammatory cytokines that reflect the level of immune response in the body. If a large number of inflammatory cytokines are released in the body, then the inflammatory response will be aggravated (44, 45). In this work, the release levels of inflammatory cytokines in mice with DSS-induced colitis were analyzed as shown in Figures 7A–C. After DSS treatment, the concentrations of TNF-α, IL-6, and IL-1β reached about 41, 350, and 50 pg/mL, drastically higher than those in the control group. Noteworthy is that the increase in inflammatory cytokine levels in the mice gavaged with fermented soymilk was significantly lower than that in the mice not treated with fermented soymilk, despite all groups receiving the same DSS treatment. The TNF-α level in the DSS + L – FSM and DSS + H – FSM groups was 21–28 pg/mL, which was close to 50% of that in the DSS treatment group. Meanwhile the level of IL-6 was 250–280 pg/mL, about 30% lower than that in the DSS group. Similarly, the concentrations of IL-1β was 25–35 pg/mL, about 50% lower than that in the DSS group. The above results indicated that the release of inflammatory factors in the serum of mice was significantly induced by the DSS treatment but was significantly inhibited by gavaging with fermented soymilk. Therefore, fermented soymilk could protect mice from colitis by inhibiting the release of inflammatory cytokines.

To elucidate the influence of fermented soymilk on oxidative stress, total antioxidant capacity (T-AOC), total superoxide dismutases (T-SOD), and glutathione peroxidase (MDA) in the serum was measured (Figures 7D–F). After DSS treatment, the concentrations of T-AOC and T-SOD were suppressed as shown in Figures 7D, E, and the level of MDA was increased in the mice with colitis compared with normal controls as shown in Figure 7F. However, the concentrations of T-AOC and T-SOD were substantially enhanced and the MDA were significantly attenuated by fermented soymilk compared with the DSS group. These results suggested that fermented soymilk can efficiently suppress the DSS-induced oxidative stress.

**Figure 8:**
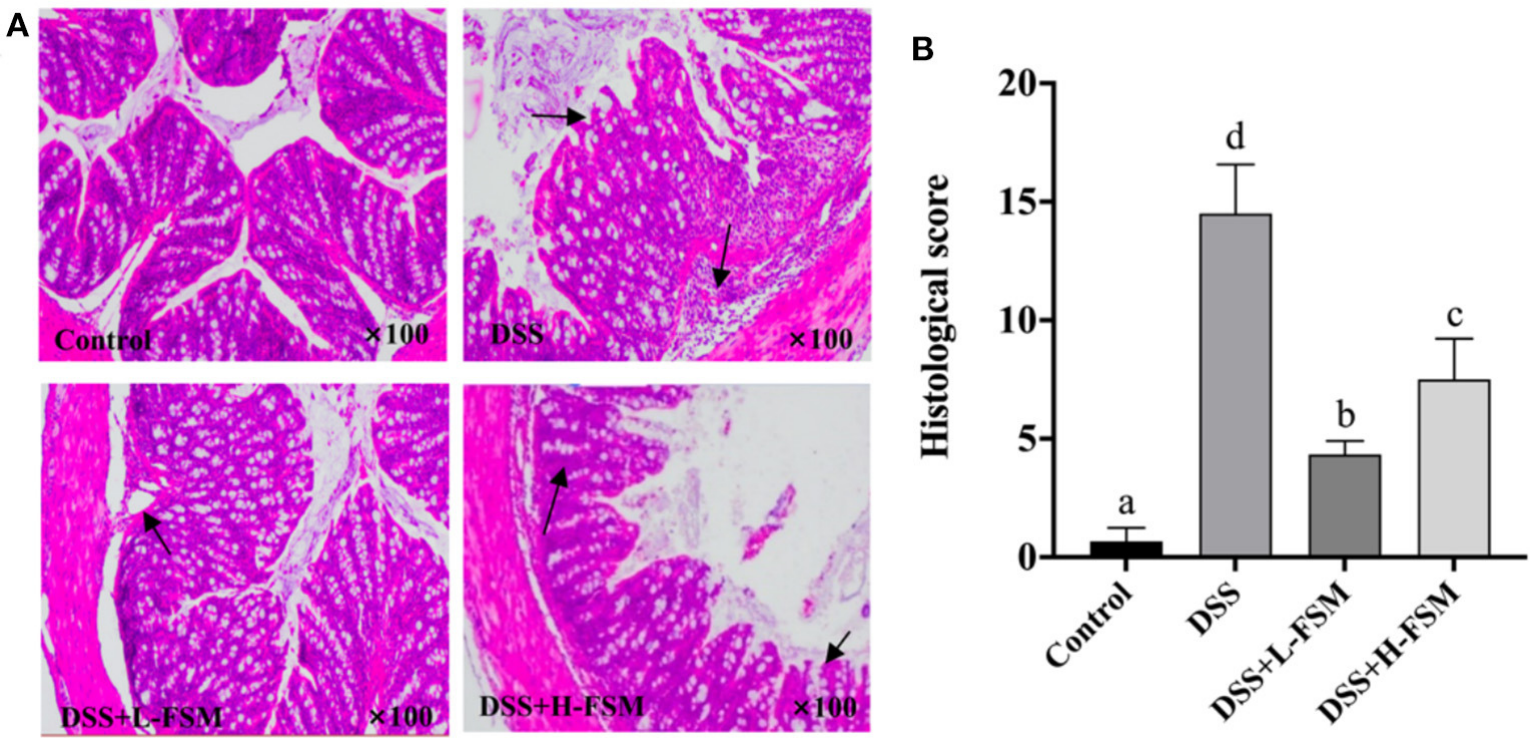
Effect of fermented soymilk on DSS-induced intestinal lesion in mice. **(A)** Histological analysis on the colonic section (*n* = 6 per group); The black arrows indicate inflammatory cell infiltration. **(B)** Histological score of the colonic section (*n* = 6 per group). (a–d) Different letters indicate significant differences between groups according to Tukey's HSD test (*p* < 0.05).

Histological analysis was conducted based on the H&E staining sections to further reveal the effect of fermented soymilk on the intestinal lesions in the mice with DSS-induced colitis. As depicted in Figure 8A, the complete mucosal surface, mucosal wrinkled wall, normal glands and crypts can be clearly observed in the control group. Although the mucosal wrinkled wall was visible after DSS treatment, the mucosal surface was incomplete, the glands were swollen and thickened, and the inflammatory cell infiltrated seriously, indicating that the animal model was successfully established. Compared with the DSS group, the mice gavaged with fermented soymilk and then treated with DSS demonstrated a better status, in which the mucosal wrinkled wall was visible, part of glands swollen and thickened, and inflammatory cell infiltrated partially. Besides, the intestinal histological score of the DSS group increased significantly to 14 (Figure 8B), while the score of the groups treated with fermented soymilk was 30–50% of that in the DSS group. This result is consistent with the analysis of inflammatory cytokine release levels in the blood of mice (Figure 7), further demonstrating the protective effect of fermented soymilk against colitis in mice.

## 4. Discussion

Recently, the antioxidant and anti-inflammatory effects of the nutrients in plant-based food have attracted extensive attention (46, 47). Fermented soymilk prepared with *Lactobacillus plantarum* HFY01 and *Lactobacillus fermentum* CQPC04 exhibits good antioxidant activity, and can significantly improve D-galactose induced premature senescence in mice (11, 12). Yang et al. (40) also found that fermented soymilk could significantly improve the antioxidant capacity of hyperlipidemia rats, and reduce the arteriosclerosis index and coronary risk index. However, the preparation process of fermented soymilk varies greatly for different production enterprises in raw materials, raw material treatment technology and strain selection. In this work, we investigated the effect of the raw material treatment, fermentation strain and fermentation time on the antioxidant activity of fermented soymilk. The results showed that all the three factors could influence the antioxidant activity of fermented soymilk, among which the hot blanching pretreatment of soybeans (BT-80 method) could improve the antioxidant activity significantly and removing the bean smell of soymilk. Furthermore, the antioxidant activity of fermented products could be improved regardless of which LAB strain was used for fermentation. Nevertheless, great differences in effect were observed between the strains. We optimized the processing conditions, including raw materials, fermentation strains and fermentation processes, and then developed fermented soymilk with high antioxidant activity for subsequent experiments.

We further studied the antioxidant effects of fermented soymilk extracts (FSE) by *in vitro* antioxidant test and using H_2_O_2_-induced Caco-2 cell injury model. *In vitro* antioxidant experiments demonstrated that fermented soymilk has better antioxidant effect than that of soymilk possibly because the fermentation process increases the content of isoflavone aglycones and phenols (48), and hydrolyzes soy protein to produce active peptides, thus enhancing the antioxidant capacity of fermented soymilk (49, 50). In this work, the results of cell experiments also showed that fermented soymilk had significant protective effect on H_2_O_2_-induced injury of Caco-2 cells. In brief, when the concentration of fermented soymilk was >0.125 mg/mL, the levels of ROS, MDA and SOD in the cells were significantly decreased (Figures 5D–F). This result is consistent with the previous reports of using fermented soymilk to protect oxidative damage in HepG2 cells (14).

Meanwhile, IBD is a chronic, progressive, and recurrent bowel disease, and one of its main causative factors is the high levels of free radicals and low antioxidant capacity (51, 52). Inhibiting lipid peroxidation and scavenging of oxygen free radicals can prevent and treat this disease (53, 54). Based on the pathogenesis of IBD, we investigated the preventive effect of fermented soymilk on DSS-induced colitis mice. Results showed that gavaged with fermented soymilk significantly improved the disease condition of DSS-induced colitis in mice. More precisely, the weight loss, the colon length, spleen-to-body ratio and disease index were improved; the release of TNF-α, IL-6, IL-8, and IL-1β was significantly inhibited; and the inflammation of colonic epithelial cells was relieved. This result further confirmed that fermented soymilk has an antioxidant and intestinal inflammation preventive effects. Considering the antioxidant effect of isoflavones, polyphenols, and active peptides in soybean and the ability of soy isoflavones and small molecule peptides to effectively relieve the symptoms of UC (55, 56), we speculated that the effect of fermented soymilk on preventing colitis in mice is closely related to the antioxidant activities of fermented soymilk. Besides, our previous work also showed that soybean β-conglycinin peptides (SGP) could promote the proliferation of Caco-2 cells, enhance the formation of intercellular tight junctions, and have a potential anti-infective ability (57). The animal experiments further revealed that the SGP could significantly reduce the histological injury, alleviate inflammatory symptoms and neutrophil infiltration in mice with DSS-induced colitis, and inhibit the expression of inflammatory regulator NF-κB/p65 (58). Moreover, LAB is a group of bacteria commonly used for the fermentation of various products, such as soymilk, milk, kimchi etc., (59, 60). LAB can regulate the balance of intestinal flora, prevent and reduce the occurrence of intestinal diseases, reduce oxidative stress damage in the intestine, and exhibit a good antioxidant activity (61, 62). The last activity can be attributed to the production of active polysaccharides, SOD, catalase (CAT) and other antioxidant enzymes, as well as thioredoxin, glutathione and other reducing substances during the growth and metabolism of LAB (63). The level of active ingredients in fermented soybean products is significantly increases during the LAB fermentation (64).

## 5. Conclusion

In summary, the antioxidant activity of fermented soymilk is significantly affected by soymilk preparation processes and fermentation time. Fermentation strain is also a key factor affecting the antioxidant activity of fermented soymilk, and great variation in antioxidant activity is observed among the soymilk samples fermented with different LAB. By exploring the differences in antioxidation of fermented soymilk, we had obtained an optimal fermentation process and used in following experiments. Cell experiments also showed that fermented soymilk could reduce the ROS and MDA contents, and increase the SOD activity in H_2_O_2_-induced Caco-2 cells to protect them from the damage of ROS and enhance their repair ability. Meanwhile, Fermented soymilk can significantly improve the disease status of DSS-induced colitis in mice by reducing the rate of weight loss, balancing the spleen-to-body weight ratio, reducing the disease activity index, and inhibiting the release of inflammatory cytokines and antioxidant index. This study provides a novel insight into the production of fermented soymilk with excellent antioxidation activity, and will facilitate the formulation of treatment and prevention strategies for IBD and other inflammatory diseases.

## Data availability statement

The raw data supporting the conclusions of this article will be made available by the authors, without undue reservation.

## Ethics statement

All experimental procedures were approved by the Animal Ethics Committee of Pony Testing International Group Co., Ltd (Approval No: PONY-2022-FL-12).

## Author contributions

YS performed the majority of the experiments and wrote the manuscript. JX, HZhao, and YL contributed to the data analysis. BY and HZhan contributed to the animal experiments. SG designed and supervised the study and checked the final manuscript. All authors contributed to the article and approved the submitted version.
